# Production performances and antioxidant activities of laying hens fed *Aspergillus oryzae* and phytase co-fermented wheat bran

**DOI:** 10.5713/ajas.20.0116

**Published:** 2020-05-12

**Authors:** Chung Ming Huang, Wen Yang Chuang, Wei Chih Lin, Li Jen Lin, Sheng Chang Chang, Tzu Tai Lee

**Affiliations:** 1 Department of Animal Science, National Chung Hsing University, Taichung 402, Taiwan; 2 School of Chinese Medicine, College of Chinese Medicine, China Medical University, Taichung 404, Taiwan; 3 Kaohsiung Animal Propagation Station, Livestock Research Institute, Council of Agriculture, Kaohsiung 912, Taiwan; 4 The iEGG and Animal Biotechnology Center, National Chung Hsing University, Taichung 402, Taiwan

**Keywords:** Phytase, *Aspergillus oryzae*, Co-fermentation, Antioxidant, Hen

## Abstract

**Objective:**

Wheat bran (WB) was co-fermented with Aspergillus oryzae and phytase (Phy) to determine whether co-fermentation improve WB phosphorus and fiber utilization in Isa-brown layers.

**Methods:**

A total of 112 Isa brown layer were randomly divided into 7 treatments with 8 replicates per a treatment and 2 hens per a replicate. The treatments included basal diet (control), basal diet supplemented with 250 unit/kg Phy (control+Phy), diet with 10% WB (10% WB), diet with 5% WB and 250 unit/kg Phy (5% WB+Phy) diet with 10% WB and 250 unit/kg Phy (10% WB+Phy), diet with 5% fermented WB supplemented with molasses and phy (PCFWH) and 125 unit/kg Phy (5% PCFWH), and diet with 10% PCFWH (10% PCFWH). The intestinal microbial population, intestinal morphology, serum antioxidant enzyme activities, and excreta phosphorus content were assessed.

**Results:**

In PCFWH, spore counts, protease activity, xylanase activity, and ferulic acid were 8.50 log/g dry matter (DM), 190 unit/g DM, 120 unit/g DM, and 127 μg/g, respectively. Xylobiose and xylotriose were released in PCFWH, while they were not detectable in WB. Antioxidant capacity was also enhanced in PCFWH compared to WB. The 10% WB+Phy and 10% PCFWH groups produced higher egg mass, but hens fed 5% WB+Phy had the lowest amount of feed intake. Eggs from 10% PCFWH had better eggshell weight, eggshell strength, and eggshell thickness. Birds fed with 10% PCFWH also had higher serum superoxide dismutase and catalase activities. Compare to control, 10% PCFWH significantly reduced excreta phosphorus content.

**Conclusion:**

Diet inclusion of 10% PCFWH improved egg quality, antioxidant status, and excreta phosphorus content of laying hens.

## INTRODUCTION

Island states like Taiwan do not have enough land area for planting to supply poultry and livestock, so domestic animal husbandry mainly relies on imported feeds. For the poultry industry, feed cost occupy about 60% to 70% of total cost of production. However, feed prices, which are depended on international prices of cereals, fluctuates over time. Elevated feed prices may have an enormous impact on national animal husbandry. Therefore, reducing feed cost and development of agricultural by-products as alternative feedstuffs are goals of recent studies.

There are a great amount of arabinoxylans in wheat bran (WB). As arabinoxylans broken down, released xylooligosaccharides are expected to promote microbial fermentation, and raise the number of lactic acid bacteria in the intestine [1]. In order to raise digestibility and utilization of nutrients in agricultural by-products, degradation of cellulose and hemicellulose are a priority. Biological treatments include enzymatic treatments and microbial fermentation, among which solid-state fermentation (SSF) is preferred to reduce the cost. Studies found that arabinoxylans from WB possessed immunomodulatory effects *in vitro* [1], while a negative effect on growth performance was shown when the WB inclusion level was over 10% in the diet of broilers [2]. It has been proved that xylooligosaccharides exert prebiotic effects, improve gut health and performance of broilers [3,4]. Accordingly, SSF of WB may not only reduce the harmful effect of non-starch polysaccharides but elevate the nutritional value of substates.

Ferulic acid is commonly found in cereals [5]. In de-starched WB, its content can reach 7.4 μg/mg [6]. Microbial enzymes were commonly used to release ferulic acid as well as feruloyl oligosaccharides (FOs) from WB dietary fiber [7]. The FOs also display great antioxidant activity *in vitro* and *in vivo* [7]. Recently, production of FOs through fermentation by *Aureobasidium pullulans* 2012 was studied and the yield of FOs could reach 774 nmol/L [8], and these FOs exert an antioxidant effect [8].

Phytic acid, also named as myo-inositol, 1,2,3,4,5,6-hexakisphosphate (IP6), is formed from an inositol ring linked with six phosphates. In WB, over 90% of total phosphorus was in form of phytate that could not be utilized as mono-gastric animals lacked endogenous phytase (Phy) [9]. Previous study indicated that *Paecilomyces variotii* fermented sorghum combined with Phy supplementation improved inorganic phosphorus content in sorghum, while whether the inoculated fungus could produce Phy was not assessed in this study [10]. Moreover, a combination of Phy addition with fungal fermentation was not compared to either treatment in this study [10]. Thus, to ensure its feasibility, the novel strategy for phosphorus release requires further exploration.

*Aspergillus oryzae (A. oryzae)* is very suitable for feed fermentation and application in animals because it can produce a tremendous amount of enzymes within short period and is categorized as Generally Recognized As Safe (GRAS) by Food and Drug Administration (GRAS Notice No. GRN 000829). Broilers fed with *A. oryzae* fermented soybean meal (FSBM) had improved growth performance and increased serum total phosphorus and IgM content in all growth stages compared with the soybean meal group [11]. Similar results were found in weaned piglets fed with *A. oryzae* FSBM [12]. Feeding FSBM improved growth performance of animals probably because the digestibility of dry matter (DM), crude protein (CP), and amino acids as well as intestinal protease activities were enhanced [12,13]. Accordingly, *A. oryzae* fermented feed has potential for animal nutrition, but little research has been carried out comparing WB with *A. oryzae* co-fermented WB with or without Phy; therefore, this research is to reveal the effect of *A. oryzae* and pytase co-fermented WB in layers diet.

## MATERIALS AND METHODS

### Solid-state fermentation

The starter culture was prepared by 30 g soybean added to 20 mL deionized water, autoclaved, inoculated with 10 mL phosphate buffered saline containing 9×10^6^ *A. oryzae* spores/mL, and incubated at 30°C for three days. The 300 g WB was mixed with 300 mL deionized water and 30 g molasses. The mixture was autoclaved at 121°C for 20 min and inoculated with the starter culture as well as 750 unit Phy. Then, the mixture was incubated at 30°C. At the end of fermentation, samples were stored at −20°C for enzyme activities and spore counts assays. In other assays, samples were dried at 50°C for 24 h, smashed, and stored at 4°C.

### Extractable phosphorus

Determination of extractable phosphorus was modified from previous study [1]. Briefly, 5 g of each sample were mixed with 50 mL 0.2% pepsin solution, which was prepared with 0.2 g pepsin (Sigma, St. Louis, MO, USA) dissolved in 100 mL 0.1 M HCl solution. After 3 h of incubation at 37°C and 80 rpm, the mixtures were centrifuged at 3,000 rpm, followed by filtration through Advantec No.1 filter papers. The 0.3 mL extracts were added to 0.6 mL deionized water and 0.6 mL vanadium-ammonium molybdate solution, which was prepared with nitric acid, ammonium metavanadate (Sigma, USA) and ammonium molybdate tetrahydrate (Sigma, USA). Then, the mixtures were centrifuged again at 6,000 rpm for 10 min, and the absorbance of suspension was detected at 415 nm. Solutions of 0.3125 to 2.5 μmol/mL potassium dihydrogen phosphate were also detected to draw a standard curve. The 300 g WB treated with 750-unit Phy was also analyzed.

### 2,2-Diphenyl-1-picrylhydrazyl scavenging activity

This assay was conducted according to previous report [14]. Samples and reagents were prepared with ethanol as the solvent. Samples were added to ethanol and vibrated for 30 min in ice bath. Mixtures were filtrated through Advantec No.1 filter paper. The filtrates were mixed with 1 mM 2,2-diphenyl-1-picrylhydrazyl (DPPH) at the ratio of 4:1, and protected from light for 30 min. Then, solutions were centrifuged at 8,000×g for 5 min, and suspensions were detected at 517 nm. Scavenging rate of DPPH was calculated by the following equation:

$$\text{Scavenging rate}=1-\frac{\text{absorbance of samples at }517\;\text{nm}}{\text{absorbance of control group at }517\;\text{nm}}$$

where ethanol was used as the control group, and butylated hydroxytoluene (BHT) was as the positive control.

### 2,2-azino-bis (3-ethyl-benzthiazoline-6-sulfonic acid) scavenging activity

The method was according to previous research [15]. Sample extraction steps were as same as in the analysis of total phenol content. The reagent was prepared with 4.4 unit/mL peroxidase, 100 μM 2,2-azino-bis (3-ethyl-benzthiazoline-6-sulfonic acid) (ABTS), and deionized water at the ratio of 1:1:6. Next, 0.1 mL extracts were mixed with 0.9 mL reagent, reacting at room temperature for 10 min. The absorbance was monitored at 734 nm, and ABTS scavenging rate was calculated using the following equation:

$$\text{Scavenging rate}=1-\frac{\text{absorbance of samples at }734\;\text{nm}}{\text{absorbance of control group at }734\;\text{nm}}$$

where deionized water was the control group, and vitamin C was as the positive control.

### Xylooligosaccharides

Xylooligosaccharides content was determined by high performance liquid chromatography (HITACHI, Kyoto, Japan) equipped with the pump (L-2130) and the RI-detector (L-2490), according to the method already set up in our laboratory [1]. Briefly, 5 g of each sample were mixed with 50 mL deionized water, and vortexed in 95°C baths for 1 h. The extracts were centrifuged at 3,000 rpm for 10 min. The suspensions were further filtrated through 0.22 μm filter paper. The temperature of column (Transgenomic CARBOSep CH0682 Pb, 300 mm×7.8 mm) was maintained at 80°C. Doubly deionized water was applied as mobile phase, and the flow rate was set at 0.4 mL/min. The standards, including D-xylose, xylobiose, xylotriose, xylotetraose, xylopentaose, and xylohexatiose, were diluted into 0.2 to 1 mg/mL for calibration.

### Experimental design

This experiment is conducted at National Chung Hsing University, Taiwan. All of the protocols for animal use were approved by the Animal Care and Use Committee (IACUC: 107-013). One hundred and twelve Isa-brown laying hens were randomly assigned to 7 treatments with 8 replicates per treatment and two hens per replicate in an individual cage (35×40×40 cm). Hens were in a house equipped with wet pad cooling system, and the temperature was controlled below 30°C. The experimental diets were fed to 29- to 40-week-old laying hens. Crumble diets and water were fed *ad libitum*. The illumination plan was conducted as 15.5 h of light and 8.5 h of darkness per day. The maize-soybean meal basal diets fed to birds were formulated to meet recommended nutrient requirements (NRC, 1994). In the first part of experiment, WB and fermented WB supplemented with molasses and phy (PCFWH) were incorporated into the diets as partial replacement for maize and soybean meal. Seven dietary treatments were as followed: basal diet (control), basal diet supplemented with 250 unit/kg Phy (control+Phy), diet with 10% WB (10% WB), diet with 5% WB and 250 unit/kg Phy (5% WB+Phy) diet with 10% WB and 250 unit/kg Phy (10% WB+Phy), diet with 5% PCFWH and 125 unit/kg Phy (5% PCFWH), and diet with 10% PCFWH (10% PCFWH). In order to equal the amount of Phy added into the diets, it was noted that 125 unit/kg Phy was added into diet with 5% PCFWH, in which Phy (250 unit/100 g WB) has been already added into PCFWH at the moment when starter culture was added. The nutrient compositions of experimental diets are shown in Table 1.

### Performance, egg production, serum, and intestinal content collection

Performance of laying hens was measured from 29- to 40-wk-old hens. Eggs weight and laying rate were recorded daily. Eggs from each replicate were collected weekly and stored at 4°C for egg quality analysis. To avoid sampling interfering with laying hen performance, other analyses were done after the record of performance was ended. Blood samples were collected at 10 a.m. from the brachial wing vein of each replicate (8 birds per treatment) using a vacuum blood collection tube. Blood samples were stored at room temperature for coagulation, and then centrifuged at 3,000 rpm for 15 min. The serum was loaded into eppendorf tubes and stored at −20°C for further analysis. For intestinal microbial population, birds were electrically stunned and dissected. Ileal and cecum contents were collected in sterilized dishes for following analysis.

### Egg quality

The egg quality was measured according to the method described by Lin et al [16]. Briefly, egg weight, eggshell strength, height of thick albumen, and yolk color were analyzed by the machine (DET 6000, NABEL Co., Ltd., Kyoto, Japan). The Haugh unit was calculated using Haugh unit formula in which height of thick albumen and egg weight were parameters. The thickness of middle section of eggshell was measured, while eggshell membrane was excluded from the shell before measurement.

### Intestinal microbial population

One gram of ileal and cecum contents was weighed, and serially diluted with sterilized PBS. Diluted samples were loaded on chromogenic medium agar (CHROMagarTM 129 ECC) and MRS medium (de Man Rogosa and Sharpe agar, Difco 288130, BD, Franklin Lakes, NJ, USA) plates for determining the population of coliform and lactic acid bacteria. The inoculated chromogenic medium plates were incubated at 37°C for 24 h, and MRS plates were incubated for 48 h. Colonies were counted and expressed as Log colony-forming unit/g of intestinal contents in four replicates.

### Intestinal morphology

After performance recording ended, hens were sacrificed by exsanguination from the jugular vein. Next, birds were dissected, and the intestines were carefully removed. Middle part of jejunum and ileum were cut out, put into the formalin, and stored at room temperature for at least one week for fixation. Fixed intestinal sections were stained using hematoxylin and eosin staining methods. The villus height, crypt depth, and the ratio of villus height to crypt depth were measured by optical microscope connected to computer software (Motic image plus 2.0, Richmond, Canada). Intestinal morphology was measured in four replicates, and ten intestinal villi and crypts per replicate were measured.

### Serum antioxidant enzymes

The superoxidase dismutase (SOD), catalase (CAT), and thiobarbituric acid reactive substances assay kits (Cayman Chemical Co., Ltd., Ann Arbor, MI, USA) were applied for determination of SOD activity, CAT activity, and total malondialdehyde (MDA) in serum samples. Serum samples were assessed in sextuplicate per treatment.

### Excreta phosphorus contents

After the record of performance and production ended, clean plastic panels were put at the bottom of cages for excreta collection. One day later, excreta from each treatment was sampled and stored at −20°C. One gram of excreta samples were put in crucibles and incinerated at 600°C for 6 h. After crucibles cooled down, 10 mL 3 N HCl was added, and boiled for at least 10 min. Then, solutions were filtrated, and quantified to 50 mL. The phosphorus content in the excreta was analyzed with molybdovanadate reagent, and different concentrations of monopotassium phosphate were prepared as standards. All treatments were analyzed in four replicates.

### Statistical analyses

Data were analyzed for significance by analysis of one-way variance and general linear model procedure of using SAS software (SAS 9.4, 2018) program. The mean value is shown in tables and figures and determination of significant differences between treatment groups was conducted using Tukey tests with p-value less than 0.05. The mathematic model was shown as follow:

$$Y_{ij}=\mu +T_{i}+{ɛ}_{ij},$$

where *Y_ij_* = results in each pen; *μ* = overall mean; *T_i_* = fixed effect of WB, Phy, and PCFWH supplementation; and *ɛ_ij_* = residual error when a pen was consider to be experimental unit, ${ɛ}_{ij}\sim N(0,\sigma_{ɛ}^{2})$.

## RESULTS

### WB and PCFWH characteristics

**Figure 1:**
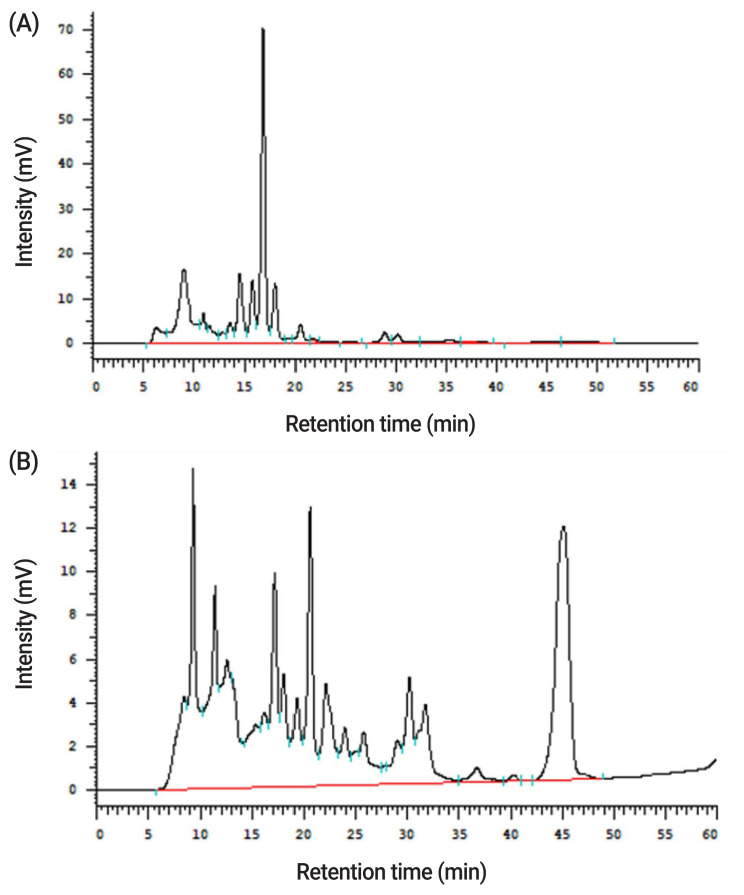
Xylooligosaccharides contents in (A) WB, and (B) PCFWH determined by high performance liquid chromatography. WB, wheat bran; PCFWH, fermented WB supplemented with molasses and phytase.

In the proximate analysis shown in Table 2, the CP, neutral detergent fiber (NDF), acid detergent fiber (ADF), and ether extract (EE) were 17.5%, 37.6%, 7.64%, and 4.35% in WB, respectively. After fermentation by *A. oryzae*, the CP, ADF, and EE content raised to 25.8%, 8.35%, and 5.13%, respectively, while NDF content decreased to 33.0% on the DM basis. Metabolites of *A. oryzae*, including spore counts, enzyme activities, and ferulic acid contents, are shown in Table 2. In WB, spore counts of *A. oryzae*, protease, and xylanase activities were not determined, while they were 8.5 log/g DM, 190 unit/g DM, and 120 unit/g DM in PCFWH, respectively. The ferulic acid contents were also measured. In PCFWH, the ferulic acid content was almost four-fold higher than that in WB (127 vs 29.0 μg/g). Results of the tyrosinase inhibition rate are shown in Table 2. Kojic acid was the tyrosinase inhibitor, so it was used as positive control. The concentrations of all examined treatments were 100 mg/mL. The tyrosinase inhibition rate of WB was not detectable, while it was 6.04% and 97.2% in PCFWH and kojic acid, respectively. Results of xylooligosaccharides contents are illustrated in Figure 1 and quantified in Table 2. All xylooligosaccharides and D-xylose were not detectable in WB. In PCFWH, D-xylose, xylobiose, and xylotriose were 9.98, 3.78, and 14.0 mg/g, respectively, while the xylotetraose, xylopentaose, and xylohexaose were not detectable.

**Figure 2:**
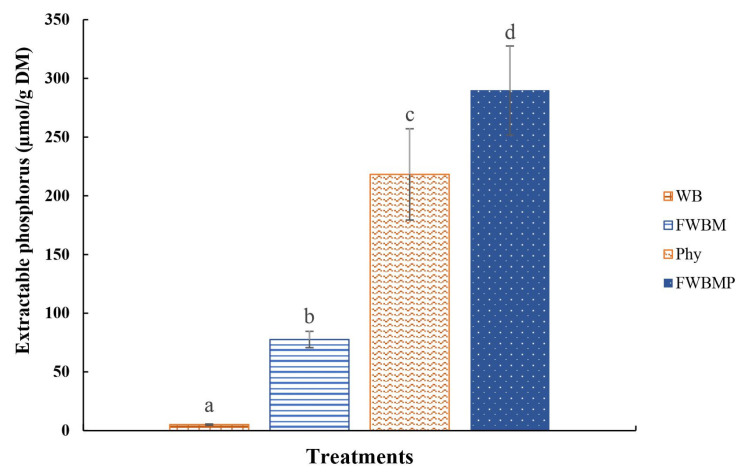
The extractable phosphorus (μmol/g dry matter) of WB with different treatments. WB, wheat bran; FWBM, fermented WB added with molasses; Phy, WB added with phytase; PCFWH, fermented WB supplemented with molasses and phytase. Each value represents the mean± tandard deviation of three replicates (n = 3). ^a–d^ Means with different letters are significantly different (p<0.05).

The extractable phosphorus was determined in acidic solutions to simulate the gastric environment and shown in Figure 2. Results demonstrated that WB rarely released inorganic phosphorus (5.09 μmol/g DM), while FWBM group had significantly enhanced inorganic phosphorus content (77.6 μmol/g DM). The WB added with Phy released 218 μmol/g DM phosphorus. Aspergillus oryzae fermented WB combined with Phy addition (PCFWH) promoted much more inorganic phosphorus (290 μmol/g DM).

**Figure 3:**
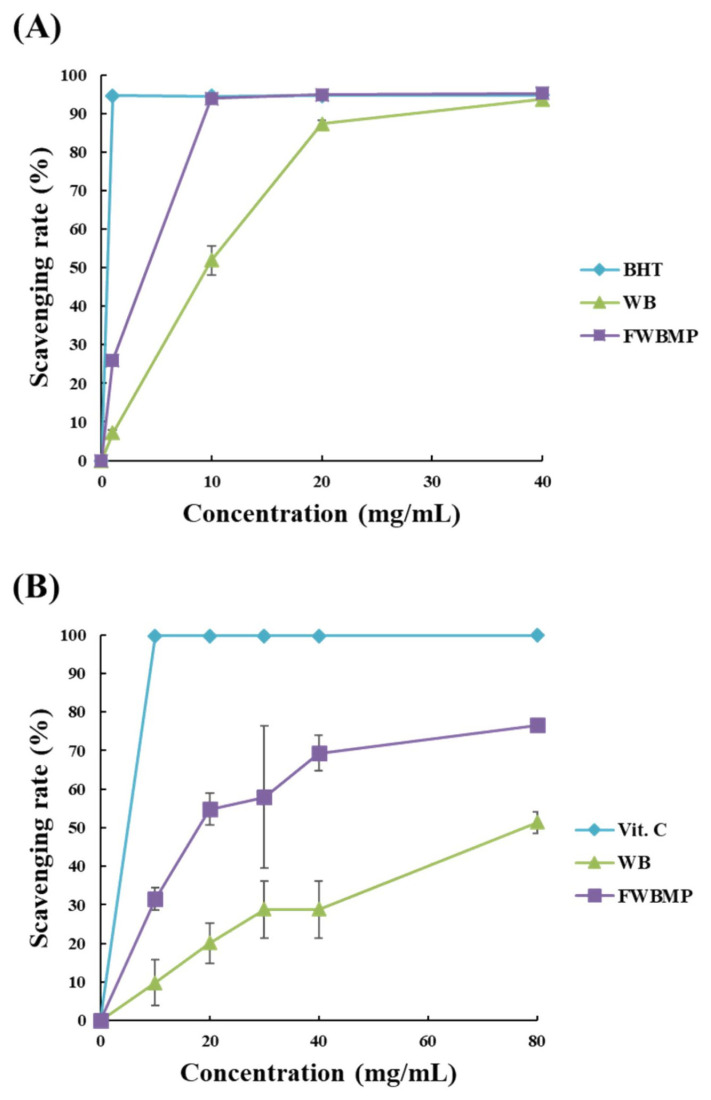
The antioxidant capacity of WB, and PCFWH at different concentrations (mg/mL) are presented as (A) 2,2-diphenyl-1-picrylhydrazyl, and (B) 2,2-azino-bis [3-ethyl-benzthiazoline-6-sulfonic acid] scavenging rate. WB, wheat bran; PCFWH, fermented WB supplemented with molasses and phytase; BHT, butylated hydroxytoluene. Each value represents the mean±standard deviation of three replicates (n = 3).

Results of *in vitro* antioxidant assays are illustrated in Figure 3 (A and B). The DPPH scavenging rate (Figure 3A) was 26.0% in PCFWH compared to 7.07% in WB at the concentration of 1 mg/mL. As the concentrations reached to 10 mg/mL, the DPPH scavenging rate of PCFWH was close to the result of BHT (94.5%) and was 41.9% higher than that of WB group (93.8% vs 51.9%). The ABTS scavenging rate (Figure 3B) was 9.85% to 51.4% at the concentrations of 10 to 80 mg/mL in WB, while it was 31.5% to 76.6% at the concentrations of 10 to 80 mg/mL in PCFWH.

### Layer performance and egg quality

Results of performance and production of laying hens from 1 to 12 wk are demonstrated in Table 3. The lowest feed intake was observed in 5% WB+Phy (p<0.05), while there was no significant difference between other treatments. The lower egg mass was found in treatments of control and 5% WB+Phy. In the results of laying rate and feed conversion ratio (FCR), no significant differences were found between treatments.

The results of egg quality analysis are shown in Table 4. The egg weight of 10% WB+Phy was significantly higher than that of control, 10% WB, control+Phy, and 5% WB+Phy. Results demonstrated that eggs of 10% PCFWH had significantly better egg quality regarding the eggshell weight, eggshell strength, and eggshell thickness (p<0.001), while control and 10% PCFWH resulted in the lowest value of yolk color compared to other treatments (p = 0.001). The heaviest egg weight occurred in 10 WB+Phy, but the eggshell strength in this treatment was the weakest. The thinnest shell thickness was found in the eggs of 5% WB+Phy. Different experimental diets did not influence the Haugh unit of tested eggs (p>0.05).

### Layer intestinal microbial population and morphology

**Figure 4:**
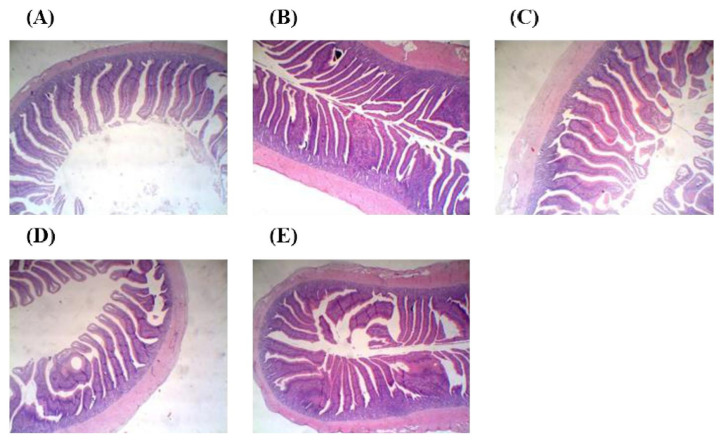
Jejunal morphology of laying hens fed experimental diets. (A) Control, (B) 5% WB+Phy, (C) 10% WB+Phy, (D) 5% PCFWH, and (E) 10% PCFWH. Control, basal diet; 5% WB+Phy, diet with 5% WB and 250 unit/kg phytase; 10% WB+Phy, diet with 10% WB and 250 unit/kg phytase; 5% PCFWH, diet with 5% PCFWH and 125 unit/kg phytase; 10% PCFWH, diet with 10% PCFWH. WB, wheat bran; Phy, WB added with phytase; PCFWH, fermented WB supplemented with molasses and phytase.

**Figure 5:**
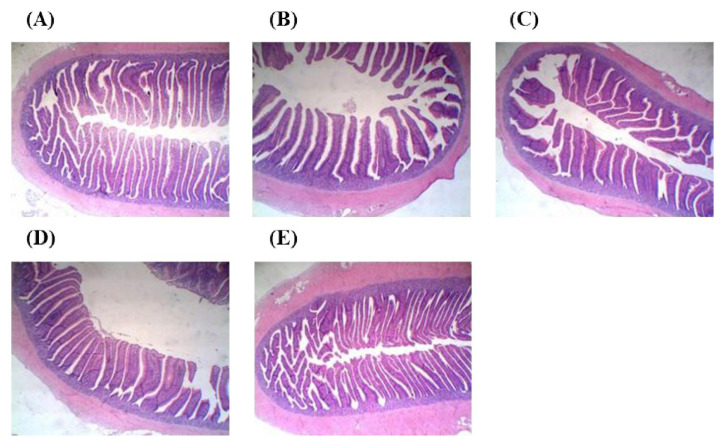
Ileal morphology of laying hens fed experimental diets. (A) Control, (B) 5% WB+Phy, (C) 10% WB+Phy, (D) 5% PCFWH, and (E) 10% PCFWH. Control, basal diet; 5% WB+Phy, diet with 5% WB and 250 unit/kg phytase; 10% WB+Phy, diet with 10% WB and 250 unit/kg phytase; 5% PCFWH, diet with 5% PCFWH and 125 unit/kg phytase; 10% PCFWH, diet with 10% PCFWH. WB, wheat bran; Phy, WB added with phytase; PCFWH, fermented WB supplemented with molasses and phytase.

Results of lactic acid bacteria and the Coliform population are shown in Table 5. No significant difference was found in the microbial populations of the ileum and cecum of laying hens between treatments (p>0.05). Results of intestinal morphology are illustrated in Figure 4 and Figure 5 and quantified in Table 6. There were no significant differences in villus height, crypt depth, and villus height to crypt depth ratio of both jejunum and ileum between treatments (p> 0.05).

### Layer serum antioxidant enzymes

Results of serum SOD, CAT, and MDA contents are demonstrated in Table 7. Experimental diets did not influence the serum MDA content (p>0.05). The 10% PCFWH treatment resulted in significantly higher serum SOD activity than other groups except for 10% WB+Phy. The highest serum CAT was also observed 10% PCFWH (p<0.05), while there was no significant difference between 5% PCFWH and 10% PCFWH.

### Layer excreta phosphorus contents

Results of excreta phosphorus contents were in Table 8. The phosphorus content in the excreta of control group was significantly higher than other treatments (p<0.05). There was no significant difference between 10% WB, control+Phy, 5% WB+Phy, 10% WB+Phy, and 10% PCFWH (p>0.05). However, the excreta phosphorus content of 5% PCFWH was higher than that in 10% PCFWH (p<0.05).

## DISCUSSION

Okot-Kotber et al [17] reveal that the Phy activities in WB varied according to the species. Normally, the Phy contained in WB is about 0.92 to 3.12 Phy unit/gram. However, they also indicate that under 60°C, the Phy activities in WB would decrease by about 80% within 1 h. Therefore, it is suggested that the difference between the results of Phy activities in WB between previous research and our research might be due to the different processing procedures. Accordingly, it is estimated that there are an extra 20 to 60 Phy unit/kg in the 10% WB supplement group and an extra 10 to 30 Phy unit/kg in the 5% WB supplement group. Co-inoculation of microorganisms and Phys has been reported, and results showed that fungal fermentation combined with Phy addition was a feasible way to elevate inorganic phosphorus in substrates [10]. Although there is high content of total phosphorus in WB, inorganic phosphorus in WB was rarely released during the 3 h extraction period. In contrast, A. oryzae fermentation combined with Phy addition released a higher level of inorganic phosphorus than either wheat bean treated with either Phys or *A. oryzae* starter culture. The Phy activity was not detectable in *A. oryzae* fermented materials, while more inorganic phosphorus was released after *A. oryzae* fermentation. Chuang et al [1] reveals that co-fermentation of probiotics and Phy can improve the inorganic phosphorus release amount because of the phytate released from complex fiber, which had been degraded by probiotics enzymes. According to the above results, the PCFWH was used in the following analysis and animal trial.

In the PCFWH treatment, the CP content increased by 8.3%, and NDF decreased 4.6% on the DM basis. Similar results were found in *A. oryzae* fermented cassava pulp where the CP content increased 9.8% and crude fiber content decreased 4% compared with non-fermented cassava pulp [18]. High spore counts, protease, and xylanase activities were also found after fermentation. It was pointed out that the content of free and linked ferulic acid in WB was 5 to 15 mg/g [19]. In the present study, the WB was extracted with deionized water, and the content of ferulic acid was only 29.0 μg/g. After fungal fermentation, it was enhanced to 127 μg/g (Table 2). During the process of fermentation, ferulic acid was released by the actions of endoxylanases and feruloyl esterases. The higher free ferulic acid content in the substrate indicated that more of the arabinoxylan structure was degraded. Accordingly, the xylooligosaccharides, as well as D-xylose contents, were increased after fermentation (Table 2). The kojic acid, the major metabolite secreted by *A. oryzae*, is known to inhibit the activity of tyrosinase. Owing to this effect, it was generally applied in cosmetics as a skin-whitening agent. It was also used for food preservation since it prevented enzymatic browning [20]. Moreover, it possesses an antioxidant as well as an immunomodulatory effect [21]. In the present study, the tyrosinase inhibition rate was detected after fermentation, proving that kojic acid might be secreted in PCFWH (Table 2). The PCFWH exerted tremendous antioxidant activities (Figure 3). The antioxidant capacity was related to the phenol content of materials. During the fermentation process, *A. oryzae* secreted proteases that degraded proteins into small peptides and amino acids, among which tyrosine has a phenolic OH-group. Secreted xylanases destroyed the arabinoxylan structure of WB and released ferulic acid as well as FOs that also displayed antioxidant properties [1]. Moreover, glucose and D-xylose in the substrate was utilized for kojic acid synthesis, indicated by the tyrosinase inhibition activity in PCFWH. All these factors contributed to the antioxidant capacity of PCFWH.

In animal trials, WB and PCFWH were incorporated into the corn-soybean diet of laying hens and broilers chickens [22,23]. Results demonstrated that hens on 5% WB+Phy treatment displayed the lowest feed intake. Hens in the 10% WB+ Phy and 10% PCFWH group produced higher egg masses, but 10% WB did not, compared to other treatments. However, there was no significant difference in FCR and laying rate between treatments. The WB is a by-product during the processing of wheat. It contains a high content of arabinoxylans that is scarcely degraded by digestive enzymes of mono-stomach animals. Therefore, the amount of WB included in the diet was limited. Comparative experiments regarding the effect of WB and fermented WB incorporated in the diet on broiler’s performance have been extensively studied, and the results indicated that broilers could endure 10% WB in the corn-soybean meal diet without displaying a decrease in performance in some experiments [24,25], however in other experiments performance was negatively affected [22, 23]. In laying hens, incorporation of 5% and 10% WB in maize-soybean meal diet did not harm the performance and production, while FCR was improved [26]. The 10% WB in the diet improved egg mass while 5% WB did not [27]. Moreover, 10% WB and Phy in the diet could replace inorganic phosphorus of layers’ diet while not exerting a negative influence on the performance and production [26]. It is well known that Phy addition can increase the phosphorus release amount from phytate thereby enhancing the phosphorus and amino acid utilization [1]. The increase of nutrient and mineral utilized efficiency would further improve the product performance of layers. These may explain why better egg mass was found in 10% WB+Phy and 10% PCFWH, but not in 10% WB in the present study. Saki et al [27] also pointed out that 10% WB in the diet did not influence feed intake, egg mass, laying rate, and FCR.

Treatments of 10% WB+Phy, 5% PCFWH, and 10% PCFWH increased egg weights compared with treatments of control, 10% WB, and control+Phy. Moreover, 10% PCFWH treatment led to the highest eggshell weight, eggshell strength, and shell thickness. It is known that egg quality is correlated to the phenolic compounds and/or flavonoids in the diets [16,28]. Yolk color is a determinative factor that impacted the preference of consumers. Yolk color is related to the xanthophyll pigments, such as carotenoids, in the diet. Lightest yolk color was observed in eggs from control group and 10% PCFWH, while the discrepancy of color score was not large (3.79 vs 4.44). Carotenoids are extensively present in legumes, such as soybean and corn [29]. In order to incorporate WB and PCFWH into diets and equal the CP and metabolizable energy of each diet, formulas for different treatments varied, so the carotenoids contents also differed depending on the amounts of different legumes used in diets. Unequal contents of carotenoids between treatments explained why the yolk color of different treatments varied. The Haugh unit, a parameter of egg protein quality, was determined by the height of thick albumen and egg weight. In the present study, there was no significant difference in the Haugh units between treatments. This is consistent with the previous report that providing *A. oryzae* culture did not change the Haugh unit [30].

Xylooligosaccharides, a kind of prebiotic, has been reported to increase bifidobacteria and total anaerobes in cecum content of rats when 5% xylooligosaccharides was in the diet [31]. A similar result was also seen in mice when 10% xylooligosaccharides was supplemented to the diet [1]. In PCFWH, xylobiose and xylotriose content was 3.78 and 14.0 mg/g, respectively. However, both diets containing 5% or 10% PCFWH did not influence the microbial population of lactic acid bacteria or coliforms in the ileum and cecum. Relatively low content of xylooligosaccharides in fermented products compared to xylooligosaccharides produced by microbial xylanases might be the reason why prebiotic properties were not observed in 5% or 10% PCFWH treatments.

The intestinal morphology is related to dietary fiber and gut health. A possible mechanism is that viscous digesta caused by dietary fiber may lead to apoptosis of villus cells, promote crypt cell proliferation, and consequently increase the villus height to crypt depth ratio [1]. It is noted that many factors, including the type of dietary fiber, ingestion period, animal species, and age, also affect intestinal morphology. In the present study, different experimental diets did not change the jejunal and ileal morphology. In contrast, 10% rapeseed meal in broilers’ diet decreased villus height and villus height to crypt depth ratio of jejunum and ileum, while 10% fermented rapeseed meal improved intestinal morphology [32]. Accordingly, the older age and more mature development of intestinal mucosa of laying hens compared to broilers may explain why intestinal morphology was rarely affected by the composition of diet.

Serum enzyme activities and MDA content were correlated to the oxidative status of animals. Environmental stressors, including high temperature, pathogens, harmful gas and enhanced oxidative pressure lead to production of free radicals and damage to the health [21]. Therefore, antioxidant feedstuff or feed additives in the diet may improve oxidative status. In the present study, free ferulic acid was released, and antioxidant capacity *in vitro* was elevated in PCFWH. Previous studies also indicated that FOs were produced in *Aureobasidium pullulans* fermented WB, and this fermented material improved serum MDA content of S180 tumor-bearing mice [8]. In the present study, the 10% PCFWH treatment elevated serum SOD, CAT activities, indicating that the oxidative status of hens was improved.

In SSF of WB, Phy was added with *A. oryzae* together to promote the release of inorganic phosphorus. In the excreta, lowest phosphorus content was seen in 10% PCFWH, but there were no significant differences between 10% WB, control +Phy, 5% WB+Phy, 10% WB+Phy, and 10% PCFWH. The 10% WB group without Phy supplementation also reduced excreta phosphorus content compared to the control group, indicating that the utilization of phosphorus in the diet increased because of intrinsic Phy content [33]. It was consistent with the previous report that 10% WB in the diet increased the concentration of serum inorganic phosphorus [26]. It was also demonstrated that the replacement of soybean meal with *A. oryzae* FSBM significantly increased serum total phosphorus content [11]. According to the result, the strategy of the combination of Phy and fungal fermentation has the potential for the reduction of phosphorus in the excreta.

## CONCLUSION

Large amounts of metabolites are present in PCFWH, including protease, xylanase, ferulic acid, xylooligosaccharides, and tyrosinase inhibition activity. Extractable phosphorus was also enhanced *in vitro*. The CP content was increased and NDF content reduced in PCFWH treatment. Furthermore, egg quality, serum SOD and CAT level, and excreta phosphorus content were improved in birds fed diet with 10% PCFWH.

## Figures and Tables

**Table 1:** Ingredients and chemical composition of the experimental diets for laying hens

Items	Control1)	10% WB	Control+Phy	5% WB+Phy	10% WB+Phy	5% PCFWH	10% PCFWH
Ingredients (g/kg)							
Corn	570.0	470.9	570.0	524.6	470.9	536.0	498.4
Soybean meal, CP 44%	216.0	134.0	216.0	180.0	134.0	170.9	97.8
WB	0	100.0	0	50.0	100.0	0	0
PCFWH	0	0	0	0	0	50.0	100.0
Phytase, unit	0	0	250	250	250	125	0
Fish meal, CP 65%	30.0	30.0	30.0	30.0	30.0	30.0	30.0
DDGS	46.9	32.0	46.9	32.0	32.0	32.0	32.0
Full fat soybean meal	12.0	95.0	12.0	52.3	95.0	50.0	110.7
Soybean oil	20.0	33.0	20.0	26.0	33.0	26.0	26.0
Limestone	73.0	73.0	73.0	73.0	73.0	73.0	73.0
Dicalcium phosphate	26.0	26.0	26.0	26.0	26.0	26.0	26.0
DL-methionine	1.30	1.30	1.30	1.30	1.30	1.30	1.30
Salt	1.50	1.50	1.50	1.50	1.50	1.50	1.50
Choline chloride	0.80	0.80	0.80	0.80	0.80	0.80	0.80
Vitamin premix2)	1.25	1.25	1.25	1.25	1.25	1.25	1.25
Mineral premix3)	1.25	1.25	1.25	1.25	1.25	1.25	1.25
Total	1,000	1,000	1,000	1,000	1,000	1,000	1,000
Calculated nutrient value							
ME (kcal/kg)	2,850	2,850	2,850	2,850	2,850	2,850	2,850
Dry matter (%)	88.5	89.0	88.5	88.8	89.0	89.1	89.7
Crude protein (%)	18.0	18.0	18.0	18.0	18.0	18.0	18.0
Crude fat (%)	5.58	8.12	5.58	6.73	8.12	6.76	7.85
Calcium (%)	3.56	3.58	3.56	3.57	3.58	3.57	3.57
Total phosphorus (%)	0.86	0.91	0.86	0.88	0.91	0.88	0.90
Available phosphorus (%)	0.68	0.70	0.68	0.66	0.70	0.66	0.66
Lysine (%)	0.93	0.98	0.93	0.97	0.98	0.94	0.93
Methionine (%)	0.46	0.46	0.46	0.46	0.46	0.45	0.45
Cysteine (%)	0.28	0.29	0.28	0.29	0.29	0.28	0.29
Phytase (unit/kg feed)4)	0	20–60	270–310	270–310	270–310	270–310	270–310
Analyzed nutrient value							
Dry matter (%)	89.2	88.9	88.8	88.5	89.0	88.7	88.9
CP (%)	18.1	18.0	18.1	18.5	17.7	18.4	18.6
Total phosphorus (%)	0.81	0.77	0.75	0.75	0.82	0.83	0.78

WB, wheat bran; Phy, WB added with phytase; PCFWH, fermented WB supplemented with molasses and phytase; CP, crude protein; DDGS, dry distillers grains with solubles; ME, metabolizable energy.

1) Control, basal diet; 10% WB, diet with 10% WB; Control+Phy, basal diet supplemented with 250 unit/kg phytase; 5% WB+Phy, diet with 5% WB and 250 unit/kg phytase; 10% WB+Phy, diet with 10% WB and 250 unit/kg phytase; 5% PCFWH, diet with 5% PCFWH and 125 unit/kg phytase; 10% PCFWH, diet with 10% PCFWH.

2) Supplied per kg of diet: Vit A 15,000 IU; Vit D_3_ 3,000 IU; Vit E 30 mg; Vit K_3_ 4 mg; riboflavin 8 mg; pyridoxine 5 mg; Vit B_12_ 25 μg; Ca-pantothenate 19 mg; niacin 50 mg; folic acid 1.5 mg; biotin 60 μg.

3) Supplied per kg of diet: Co (CoCO_3_) 0.255 mg; Cu (CuSO_4_·5H_2_O) 10.8 mg; Fe(FeSO_4_·H_2_O) 90 mg; Zn (ZnO) 68.4 mg; Mn (MnSO_4_·H_2_O) 90 mg; Se (Na_2_SeO_3_) 0.18 mg.

4) The activities unit of phytase presented by phytase unit (FTU) per kg feed and the activities of phytase was estimated according to the addition amount and research of Okot-Kotber et al [17].

**Table 2:** Proximate analysis of wheat bran and fermented wheat bran supplemented with molasses and phytase

Items	WB	PCFWH
DM	88.1±0.20	94.8±0.13
CP (% DM)	17.5±0.53	25.8±0.67
NDF (% DM)	37.6±0.74	33.0±0.41
ADF (% DM)	7.64±0.17	8.35±0.35
EE (% DM)	4.35±0.82	5.13±0.09
Ash (% DM)	5.11±0.31	6.97±0.05
Spore counts (log/g DM)	ND	8.50±0.27
Protease (unit/g DM)1)	ND	190±16.4
Xylanase (unit/g DM)2)	ND	120±13.9
Ferulic acid (μg/g)	29.0±12.4	127±23.1
D-xylose (mg/g)	ND	9.98±0.50
Xylobiose (mg/g)	ND	3.78±0.21
Xylotriose (mg/g)	ND	14.0±0.84

All data are expressed as means±standard deviation from three replicates (n = 3).

WB, wheat bran; PCFWH, fermented WB supplemented with molasses and phytase; DM, dry matter; CP, crude protein; NDF, neutral detergent fiber; ADF, acid detergent fiber; EE, ether extract; ND, not determined.

1) One unit of protease activity is defined as 1 μg L-tyrosine is generated from 10 mg/mL casein in the condition of 40°C and pH 7.5 in a minute.

2) One unit of xylanase activity is defined as 1 μmol D-xylose is generated from 10 mg/mL xylan in the condition of 37°C and pH 5.5 in a minute.

**Table 3:** Effects of experimental diets on the performance and production of laying hens from 1 to 12 wk

Items	Experimental diets1)							SEM	p-values

	Control	10% WB	Control+Phy	5% WB +Phy	10% WB+Phy	5% PCFWH	10% PCFWH		
Feed intake (g/d/bird)	108^b^	104^ab^	105^ab^	99.9^a^	109^b^	111^b^	108^b^	2.25	0.022
Laying rate (%)	91.3	93.5	92.2	91.6	93.3	93.4	95.8	1.12	0.104
Egg mass (g/d/bird)	52.2^a^	52.8^ab^	52.5^ab^	51.94^a^	55.1^b^	54.5^ab^	55.0^b^	0.85	0.025
FCR (%)	2.06	1.97	2.00	1.92	1.98	2.03	1.98	0.03	0.063

Each value represents the mean eight replicates from 1 to 12 wk.

WB, wheat bran; Phy, WB added with phytase; PCFWH, fermented WB supplemented with molasses and phytase; SEM, standard error of the mean; FCR, feed conversion ratio.

1) Control, basal diet; 10% WB, diet with 10% WB; Control+Phy, basal diet supplemented with 250 unit/kg phytase; 5% WB+Phy, diet with 5% WB and 250 unit/kg phytase; 10% WB+Phy, diet with 10% WB and 250 unit/kg phytase; 5% PCFWH, diet with 5% PCFWH and 125 unit/kg phytase; 10% PCFWH, diet with 10% PCFWH.

a,b Means with in the same row with different letters are significantly different (p<0.05).

**Table 4:** Effects of experimental diets on the egg quality from 1 to 12 wk

Items	Experimental diets1)							SEM	p-value

	Control	10%WB	Control +Phy	5%WB+Phy	10%WB +Phy	5% PCFWH	10% PCFWH		
Egg weight (g)	56.3^a^	56.9^a^	57.0^a^	57.4^ab^	58.8^c^	58.6^bc^	58.7^bc^	0.44	<0.001
Eggshell weight (g)	6.55^ab^	6.60^bc^	6.58^bc^	6.39^a^	6.75^cd^	6.73bcd	6.83^d^	0.06	<0.001
Eggshell strength (kg/cm^2^)	4.17abc	4.15abc	4.29bcd	4.07^ab^	4.04^a^	4.36^cd^	4.51^d^	0.07	<0.001
Shell thickness (mm)	0.36^ab^	0.37bcd	0.36^bc^	0.35^a^	0.36^bc^	0.37^cd^	0.37^d^	0.003	<0.001
Yolk color	3.85^a^	4.00^ab^	4.19^bc^	4.44^c^	4.29^bc^	4.04^ab^	3.79^a^	0.11	0.001
Haugh unit	81.5	78.9	78.9	80.2	80.4	79.5	80.4	0.62	0.058

Each value represents the mean of six replicates.

WB, wheat bran; Phy, WB added with phytase; PCFWH, fermented WB supplemented with molasses and phytase; SEM, standard error of the mean.

1) Control, basal diet; 10% WB, diet with 10% WB; Control+Phy, basal diet supplemented with 250 unit/kg phytase; 5% WB+Phy, diet with 5% WB and 250 unit/kg phytase; 10% WB+Phy, diet with 10% WB and 250 unit/kg phytase; 5% PCFWH, diet with 5% PCFWH and 125 unit/kg phytase; 10% PCFWH, diet with 10% PCFWH.

a–d Means with in the same row with different letters are significantly different (p<0.05).

**Table 5:** Effects of experimental diets on microbial population in the ileum and cecum of laying hens

Items	Experimental diets1)					SEM	p-value

	Control	5% WB+Phy	10% WB+Phy	5% PCFWH	10% PCFWH		
Lactic acid bacteria (log CFU/g)							
Ileum	8.08	8.40	8.48	8.74	8.21	0.39	0.800
Cecum	9.10	9.03	9.24	9.28	8.97	0.19	0.765
Coliform (log CFU/g)							
Ileum	2.90	3.76	3.37	3.15	3.00	0.48	0.809
Cecum	6.33	6.61	6.12	7.41	6.82	0.33	0.103

Each value represents the mean of four replicates.

WB, wheat bran; Phy, WB added with phytase; PCFWH, fermented WB supplemented with molasses and phytase; SEM, standard error of the mean; CFU, colony-forming unit.

1) Control, basal diet; 5% WB+Phy, diet with 5% WB and 250 unit/kg phytase; 10% WB+Phy, diet with 10% WB and 250 unit/kg phytase; 5% PCFWH, diet with 5% PCFWH and 125 unit/kg phytase; 10% PCFWH, diet with 10% PCFWH.

**Table 6:** Effect of experimental diets on the intestinal morphology of laying hens

Items	Experimental diets1)					SEM	p-value

	Control	5% WB+Phy	10% WB+Phy	5% PCFWH	10% PCFWH		
Jejunum							
Villus height (μm)	1,168	1,139	1,124	1,011	1,054	80.6	0.641
Crypt depth (μm)	162	170	166	145	134	11.2	0.161
Villus height/crypt depth	7.25	6.70	6.82	7.08	7.87	0.43	0.366
Ileum							
Villus height (μm)	888	876	884	821	758	46.5	0.269
Crypt depth (μm)	123	143	137	127	123	10.5	0.612
Villus height/crypt depth	7.28	6.29	6.54	6.50	6.23	0.48	0.556

Each value represents the mean of four replicates.

WB, wheat bran; Phy, WB added with phytase; PCFWH, fermented WB supplemented with molasses and phytase; SEM, standard error of the mean.

1) Control, basal diet; 5% WB+Phy, diet with 5% WB and 250 unit/kg phytase; 10% WB+Phy, diet with 10% WB and 250 unit/kg phytase; 5% PCFWH, diet with 5% PCFWH and 125 unit/kg phytase; 10% PCFWH, diet with 10% PCFWH.

**Table 7:** Effect of experimental diets on the serum antioxidant enzyme activities and malondialdehyde content of laying hens

Items	Experimental diets1)					SEM	p-value

	Control	5%WB+Phy	10% WB +Phy	5% PCFWH	10% PCFWH		
MDA (μM)	55.2	61.1	56.7	64.4	62.3	3.55	0.336
SOD (U/mL)	40.8^a^	39.2^a^	42.0^ab^	39.4^a^	44.9^b^	1.01	0.003
CAT (nmol/min/mL)	79.7^a^	75.2^a^	79.4^a^	108^ab^	157^b^	23.5	0.022

Each value represents the mean of six replicates.

WB, wheat bran; Phy, WB added with phytase; PCFWH, fermented WB supplemented with molasses and phytase; SEM, standard error of the mean; MDA, malondialdehyde; SOD, superoxidase dismutase; CAT, catalase.

1) Control, basal diet; 5% WB+Phy, diet with 5% WB and 250 unit/kg phytase; 10% WB+Phy, diet with 10% WB and 250 unit/kg phytase; 5% PCFWH, diet with 5% PCFWH and 125 unit/kg phytase; 10% PCFWH, diet with 10% PCFWH.

a,b Means with in the same row with different letters are significantly different (p<0.05).

**Table 8:** Effect of experimental diets on the excreta P contents

Item	Experimental diets1)							SEM	p-value

	Control	10% WB	Control +Phy	5% WB +Phy	10% WB +Phy	5% PCFWH	10% PCFWH		
Excreta P (%)	1.79^a^	0.98^bc^	1.16^bc^	0.89^bc^	1.08^bc^	1.26^b^	0.74^c^	0.14	0.002

Each value represents the mean of four replicates.

WB, wheat bran; Phy, WB added with phytase; PCFWH, fermented WB supplemented with molasses and phytase; SEM, standard error of the mean.

1) Control, basal diet; 10% WB, diet with 10% WB; Control+Phy, basal diet supplemented with 250 unit/kg phytase; 5% WB+Phy, diet with 5% WB and 250 unit/kg phytase; 10% WB+Phy, diet with 10% WB and 250 unit/kg phytase; 5% PCFWH, diet with 5% PCFWH and 125 unit/kg phytase; 10% PCFWH, diet with 10% PCFWH.

a–c Means with in the same row with different letters are significantly different (p<0.05).
